# A prospective randomised trial comparing nasogastric with intravenous hydration in children with bronchiolitis (protocol) The comparative rehydration in bronchiolitis study (CRIB)

**DOI:** 10.1186/1471-2431-10-37

**Published:** 2010-06-01

**Authors:** Ed Oakley, Franz E Babl, Jason Acworth, Meredith Borland, David Kreiser, Jocelyn Neutze, Theane Theophilos, Susan Donath, Mike South, Andrew Davidson

**Affiliations:** 1 Department of Emergency Medicine Monash Medical Centre, Clayton Rd, Clayton Victoria 3168 Australia; 2 Murdoch Children's Research Institute Flemington Rd Parkville Victoria 3052 Australia; 3 Southern Clinical School, Faculty of Medicine, Nursing and Health Sciences, Monash University, Victoria 3800 Australia; 4 Department of Emergency Medicine Royal Children's Hospital, Flemington Rd, Parkville, Victoria 3052, Australia; 5 Department of Paediatrics, Faculty of Medicine, Dentistry and Health Sciences, University of Melbourne, Victoria 3010 Australia; 6 Department of Emergency Medicine Royal Children's Hospital Brisbane, Herston Rd, Herston Queensland 4006 Australia; 7 Department of Emergency Medicine Princess Margaret Hospital Roberts Rd, Subiaco, Perth 6008 Western Australia; 8 Department of Emergency Medicine, Sunshine Hospital 176 Furlong Rd, St Albans Victoria 3021 Australia; 9 Department of Emergency Medicine Kidz First Hospital, 100 Hospital Road, Papatoetoe, Auckland 2025, NZ; 10 Department of Medicine Royal Children's Hospital Flemington Rd, Parkville Victoria 3052 Australia; 11 Department of Anaesthesia, Royal Children's Hospital, Flemington Rd, Parkville, Victoria 3052 Australia

## Abstract

**Background:**

Bronchiolitis is the most common reason for admission of infants to hospital in developed countries. Fluid replacement therapy is required in about 30% of children admitted with bronchiolitis. There are currently two techniques of fluid replacement therapy that are used with the same frequency-intravenous (IV) or nasogastric (NG).

The evidence to determine the optimum route of hydration therapy for infants with bronchiolitis is inadequate. This randomised trial will be the first to provide good quality evidence of whether nasogastric rehydration (NGR) offers benefits over intravenous rehydration (IVR) using the clinically relevant continuous outcome measure of duration of hospital admission.

**Methods/Design:**

A prospective randomised multi-centre trial in Australia and New Zealand where children between 2 and 12 months of age with bronchiolitis, needing non oral fluid replacement, are randomised to receive either intravenous (IV) or nasogastric (NG) rehydration.

750 patients admitted to participating hospitals will be recruited, and will be followed daily during the admission and by telephone 1 week after discharge. Patients with chronic respiratory, cardiac, or neurological disease; choanal atresia; needing IV fluid resuscitation; needing an IV for other reasons, and those requiring CPAP or ventilation are excluded.

The primary endpoint is duration of hospital admission. Secondary outcomes are complications, need for ICU admission, parental satisfaction, and an economic evaluation. Results will be analysed using t-test for continuous data, and chi squared for categorical data. Non parametric data will be log transformed.

**Discussion:**

This trial will define the role of NGR and IVR in bronchiolitis

**Trail registration:**

The trial is registered with the Australian and New Zealand Clinical Trials Registry - ACTRN12605000033640

## Background

Bronchiolitis is a disease of the lower respiratory tract with peak incidence in the winter. It is the leading cause of hospitalisation during the first year of life and is a major cause of morbidity and mortality [1-5]. Victorian state-wide data for the calendar year 2006 reveal 2280 admissions for bronchiolitis, with an average length of stay of 3.2 days. One hundred sixty eight (7.4%) patients were admitted to intensive care. The estimated cost of the Victorian bronchiolitis hospital admissions for 2006 is $8.1 million dollars (data supplied at personal request from Victorian Admitted Episodes Dataset, Performance Reporting & Analysis Unit, Department of Human Services Victoria). In the United States of America bronchiolitis accounts for 21% of all hospital admissions for children under 1 year of age, with an annual cost of $390 million dollars [5].

There are no clearly effective chemotherapeutic treatments to improve the outcome of infants with bronchiolitis, so treatment is limited to oxygen, supportive care of breathing, and fluid replacement therapy[1,2,6-10]. Maintaining hydration is an important component of the care of infants with bronchiolitis. Fluid replacement therapy is required in about 30% of children admitted with bronchiolitis, due to reduced oral intake and evaporative losses from increased respiratory effort and fever [11].

There are currently two techniques of fluid replacement therapy, IV or NG. There is a lack of agreement on which method of fluid replacement is most beneficial for patients with bronchiolitis - we do not know which therapy is better. They both have theoretical grounds for superiority, but both also have a range of possible complications. As fluid replacement is such an important aspect of the management of this common disease it is very important that fluid therapy is evidence based.

Nasogastric fluid replacement (NGR) is effective and has few complications. It provides the physiological benefit of enteral hydration, which allows the body to absorb the required amounts of water and solute. In children with dehydration due to gastroenteritis, NGR is the treatment of choice as it provides faster recovery, shorter hospital stay and lowers the cost compared to IV fluid replacement (IVR) [12,13]. A nasogastric tube (NGT) can often be inserted more easily than an IV catheter, especially in infants with poor peripheral circulation due to reduced oral intake. However, concerns have been raised regarding the use of nasogastric tubes (NGT) in infants with bronchiolitis. There may be an increased risk of aspiration in patients with bronchiolitis [14]. The partial obstruction of the upper airway may compromise respiratory function, [15-18] with some studies showing up to a 50% increase in the work of breathing [16]. However these studies have been mostly in young or preterm infants and the ability to extrapolate these findings to older infants with bronchiolitis is questionable.

Intravenous fluid replacement (IVR) is commonly used in bronchiolitis[1]. However, accidental water intoxication and electrolyte imbalances are potential complications [10]. Up to 30% of children admitted with bronchiolitis have been shown to have some degree of hyponatraemia requiring vigilance with IV fluid administration[19,20]. It has also been postulated that delaying enteral nutrition will cause delay in recovery due to loss of nutritional status, and that the persisting hunger may make the children more irritable.

Kennedy et al (2005)[21] reviewed the evidence for fluid replacement in children with bronchiolitis. The authors report a wide variation in practice, with use of NGR in many units. They conclude that "there is no good quality evidence for or against the use of NGR in infants with bronchiolitis. A randomised controlled trial is needed." Vogel (2003) [22] described the management of bronchiolitis admissions in New Zealand hospitals in 1998. There was a large variation in management between hospitals, with between 6% and 64% of children needing fluid replacement therapy receiving NGR.

We confirmed the wide variation of practice in Australia with a pilot study. As baseline data for our proposed study, a survey of Australasian paediatric emergency physicians was undertaken in 2005, by the Paediatric Research in Emergency Departments International Collaborative (PREDICT), a research collaborative of all tertiary paediatric emergency departments and major mixed emergency departments in Australia and New Zealand). Of the 78 senior doctors surveyed at 11 sites (including all tertiary paediatric emergency departments in Australia and New Zealand), 48% reported using NGR and 52% IVR as initial treatment in acute bronchiolitis. There were no guidelines in any institution to determine which fluid replacement therapy should be used in bronchiolitis [23].

There are no published trials directly comparing NGR and IVR. The research available on NGR in bronchiolitis comes from two small studies. In a case series of 37 infants with bronchiolitis, Sammartino (2002) [24] found NGR was tolerated without incident. Two infants deteriorated as the illness progressed and removal of the NGT did not help.

Smyth and Openshaw[10] in a *Lancet *review article conclude "Neither oxygen therapy, nor fluid replacement strategies have been validated in large randomised controlled trials, although the dangers of hyponatraemic fluid overload are recognised. These treatment options should be addressed in randomised controlled trials." Further Smyth states that "Clinical trials of interventions in bronchiolitis have been criticised for being too small and focusing on short-term outcomes, rather than reporting outcomes of interest to clinicians and parents, such as length of hospital stay."

Davison et al [6] conclude in another recent review: "There is an ongoing need for studies that are adequately powered to identify therapies that may benefit critically ill children with this disease (bronchiolitis). Because the mortality associated with bronchiolitis is low, large multicentre trials that focus on continuous outcome measures such as duration of mechanical ventilation and ICU or hospital length of stay will provide the best opportunity to demonstrate a clinical benefit with adequate statistical power."

The evidence to determine the optimum route of hydration therapy for infants with bronchiolitis is inadequate. This randomised trial will be the first to provide good quality evidence of whether NGR offers benefits over IVR.

## Methods/Design

### Study Aims

The primary aim of this multicentre randomised trial is to investigate whether the type of fluid replacement - NG versus IV - affects the duration of hospital admission, in children aged between 2 and 12 months admitted to hospital with a clinical diagnosis of bronchiolitis. Secondary aims are to undertake an economic appraisal, and to determine the difference in the incidence of complications, duration of treatment and parental satisfaction for the two treatments.

We hypothesise that there will be a difference in the length of hospital stay between the two treatment modalities.

### Study Design and setting

This study is an open randomised trial comparing two interventions. This study will take place in 6 paediatric emergency departments and inpatient wards in Australia and New Zealand.

### Ethical considerations

The study has ethical approvement at all participating sites. All parents/guardians are provided with both verbal and written information about the study and written informed consent is obtained prior to enrolment in the trial.

The trial is overseen by a trial steering committee that is responsible for the ethical and rigorous conduct of the trial.

A data monitoring committee (with membership entirely independent of the conduct of the trial) will monitor adverse events and review outcomes and complications annually. They will report and make recommendations to the trial steering committee. No formal stopping rules are in place.

### Subject Selection

**Figure 1:**
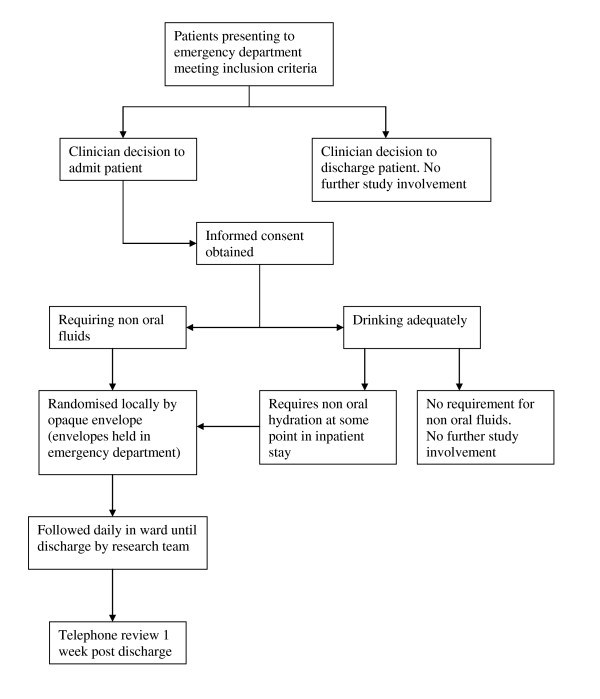
**Patient identification and entry into CRIB study**.

Children satisfying the inclusion and exclusion criteria who are admitted to the hospital will be eligible for the study. Patients admitted to the ward with bronchiolitis who deteriorate and need institution of hydration will be eligible for randomisation. Figure 1 outlines the flow of patients through the study.

### Definition of Disease State

The eligible patients will be all children younger than 12 months and older than 8 weeks (corrected for prematurity) presenting to the Emergency Department with bronchiolitis requiring hydration. The need for hydration will be defined by a history of oral intake less than 50% in the last 6 hours or signs of dehydration such as reduced urine output, tachycardia (in the absence of fever), dry mucous membranes, decreased skin turgor or sunken fontanelle.

### Inclusion and Exclusion Criteria

Inclusion and exclusion criteria are shown in Additional File 1. In order to keep the study group as homogeneous as possible patients younger than 8 weeks were excluded as the very young often have a different and more severe clinical presentation and course of bronchiolitis. Children older than 12 months were also excluded as other diagnoses, in particular asthma assume an increasing role, impacting on the identification and management of these patients.

### Patient randomisation

The patients will be randomised at the time they require non oral fluid replacement. Randomisation will be stratified by 2 age groups (2 months to less than 6 months, 6 months to less than 12 months) and by site. Randomisation will be blocked in 3 randomly allocated block sizes to ensure concealment of allocation. Each site will have its own set of opaque sealed randomisation envelopes. These will have a reminder of inclusion and exclusion criteria to be assessed before the envelope is opened.

### Randomised treatments

#### Intravenous fluid replacement group

Topical anaesthetic cream will be used in accordance with hospital policy to reduce the pain of IV insertion. Insertion of the IV cannula will be at the site of anaesthetic cream application after 30-60 minutes. Size 22 or 24 gauge cannulae will be used for all cases. The fluid used for hydration will be 0.45% Sodium Chloride with 2.5 or 5% dextrose. Ongoing fluid replacement will be guided by the measured serum electrolytes (see "Data Collection").

#### Nasogastric fluid replacement group

Insertion of the NGT will be by experienced personnel. The nostril will be sprayed with local anaesthetic spray 2 minutes prior to NGT insertion. The tube will be measured from nares to epigastrium to determine depth of insertion. Post insertion correct tube placement will be confirmed by aspiration of stomach contents with pH < 4. Failed aspiration or pH ≥4 should be repeated after 30 minutes. A repeated failure to confirm the presence of the NGT in the stomach by these means requires an abdominal/chest x-ray to be performed to confirm the position of the tube prior to its use. If there is doubt about the position of the tube after this it should be removed and replaced. Tubes will be size: 6 French < 6 months of age, and 8 French 6-12 months of age. NGR will be by continuous infusion with a standard oral electrolyte solution for the first two hours, followed by expressed breast milk or formula.

##### For both groups

All patients will be admitted to the ward under the appropriate unit. Total fluid replacement will not exceed 100% of normal daily requirements. Oral feeds will be at the discretion of the clinical team and the IV or NG fluid rate adjusted accordingly.

A parent satisfaction score will be obtained using a standardised, five point Likert scale. The score will be obtained daily from one parent or by agreement with both parents if they are in attendance. A final "overall" satisfaction score will be obtained immediately prior to hospital discharge.

All patients will be followed up at 5 to 7 days post discharge with a standard questionnaire to determine the incidence of complications, or need for further medical care.

### Study Procedure

All patients will have all clinical observations entered into a Clinical Report Form (CRF) by the nursing staff at the time of performing the observations. All CRFs will be checked daily by research staff while the participant is an inpatient.

#### Clinical observations from nursing records

SpO_2_, respiratory rate, respiratory effort, and heart rate will be measured at enrolment, at hourly intervals for the first 2 hours then 2 hourly. These will allow comparison of groups by severity of disease at enrolment, and ensure any clinically relevant complications are captured.

Oxygen administered (L/min for nasal cannulae or %O_2 _for head box) will be documented for each shift. The times oxygen is commenced and ceased will be documented. This will allow accurate capture of the duration of oxygen therapy. Type, route and volume of fluid administered will be recorded, with hourly, shift, and daily totals. This allows assessment of fluid requirements and cross referencing volumes of fluid infused. Hydration status of the patients will be measured using urine output (with shift and daily totals) and weight (measured at the time of admission, and then daily).

Complications recorded will include: episodes of apnoea and bradycardia (lasting more than 20 seconds); all episodes where the SpO_2 _was recorded at < 90%; and the need for replacement of, or problems with the IV cannula or NGT.

The need for and duration of ICU admission will be documented. Duration of hospital admission will be documented and checked by calculation from the hospital admissions and discharge system.

#### Laboratory investigations

Electrolytes, urea and creatinine, and glucose levels will be measured when inserting the IV line. Electrolytes will be repeated 6-8 hours after commencement of therapy in the intravenous therapy group (as an accepted standard in IV fluid management); and daily thereafter. Blood sampling may be by draw back on IV cannulae with discarding of 2 mls of blood or alternatively sampling by IV access or capillary blood sample at the discretion of physician managing patient. All testing of electrolyte levels in patients with iv fluids and non testing in patients with NG fluids are in line with current local recommended practice and represent standard care at the participating hospitals [23]. No additional blood tests will taken for the purpose of this trial.

Other investigations will be at the discretion of the managing physician.

#### Post discharge phone review

Will be undertaken for all patients 5 to 7 days post discharge. Information collected will include return visits to a hospital, admission to hospital, attendance at local medical officer - planned and unplanned and other complications including difficulty feeding, nasal sores, arm bruising or sores.

### Guidelines for oxygen therapy

• Oxygen should be commenced to keep SpO2 ≥ 92%, or if there is markedly increased work of breathing and respiratory distress.

• The least amount of oxygen needed to keep SpO2 ≥ 92% should be administered.

• Oxygen should be ceased when SpO2 is consistently above 92%, and the child is able to feed well. Cease by weaning the flow as tolerated until 1 l/min or less is needed.

Guidelines for non-oral fluid commencement are presented in Table 1.

**Table 1:** Guidelines for commencement of non oral fluid therapy.

Weight (Kg)	Fluid rate (mls/hr)	
	**Intravenous**	**Nasogastric**

3.0	10	12
4.0	13	18
5.0	16	21
6.0	19	24
7.0	22	27
8.0	26	30
9.0	29	33
10	32	36
12	35	39
15	40	42
20	48	50

**Note: **total initial hydration rate is determined at about 80% maintenance fluid requirements, and should never exceed 100% of daily fluid requirements

Guidelines for changing the treatment from one intervention to the other are presented in Additional File 2.

### Outcome measures

The primary outcome measure is the inpatient length of stay. This will be recorded in 2 ways: 1) the actual duration of inpatient stay will be determined from the computerised database of patient admission; 2) Given that the length of stay can be affected by many administrative and social factors unrelated to the condition of the child, we will record and report also the time until the child is ready for discharge using objective criteria. An infant will be considered ready for discharge if he/she has not received supplemental oxygen for 12 hours, has had stable respiratory status for 4 hours (including slight or nil recession) and is feeding adequately.

The secondary outcome measures are the incidence and type of complications of hydration therapy (complications will include any adverse event related to the method of hydration, including but not limited to electrolyte abnormalities, IV line/NGT needing replacement, infection, thrombosis of the IV site, trauma to the nose from the NGT, pulmonary aspiration, extravasation of IV fluid); the duration of therapy; parental satisfaction (measured daily) and an economic appraisal of each intervention.

### Sample size, power and statistical methods

The minimal clinically significant difference in length of hospital stay was thought to be greater than or equal to 12 hours. In order to perform the sample size calculation we determined mean duration of hospital stay and standard deviation (sd) from two available sets of admitted patients with bronchiolitis. Mean (sd) length of inpatient stay in a retrospective review of 36 patients admitted with bronchiolitis and requiring fluid replacement was 84 (49) hours. For the patients who were enrolled and randomised in the pilot study, mean (sd) duration of admission was 98 (70) hours, suggesting that patients who enter the study may have more severe illness.

Recruiting a total of 750 subjects (375 per arm) will give 80% power to detect an effect size of 0.2 (a small effect) using a two group t-test with a 0.05 two-sided significance level. That is a difference of 0.2 standard deviations between the mean length of stay in the 2 groups. If the sd is 70 hours (as in the pilot of the protocol), the study will be able to detect a difference between the groups of 14 hours; if the sd is 50 hours (as in the retrospective review), the study will be able to detect a difference of just over 8 hours. Thus, this study is powered to detect a difference in duration of admission of 14 hours, and possibly much less.

For the secondary outcomes, using this sample size, and knowing that approximately 30% of all intravenous cannulae have been shown to have complications,[25-28] gives 80% power to detect a 30% change in complications (from 30% to 20%) with a 0.05 significance level.

Analysis will be by intention to treat. Data will be analysed descriptively and statistically using the STATA data analysis program. Since this is a RCT and 2 of the outcome measures are measured on a continuous scale (duration of inpatient stay or hours of therapy), t-tests will be used to compare the 2 groups. If necessary due to skewed data, the data will be log-transformed. Data on parental satisfaction and complications will be treated as categorical and a Chi-squared test will be used.

No interim analysis of the data is planned.

### Economic evaluation

A trial-based economic evaluation will be conducted to assess which approach to fluid replacement therapy is the more 'cost-effective', as judged by their 'cost per child ready for discharge' ratio. The appraisal will be a cost-effectiveness analysis (i.e. outcomes measured with clinically meaningful indicators) using decision tree analysis. The choice of Cost Effective Analysis reflects the research question - one of 'technical efficiency' (i.e. which treatment approach to use) rather than 'allocative efficiency' (i.e. whether treatment should be provided). The appraisal will be conducted from a 'health sector perspective', but with a primary focus on the 'hospital as provider' and 'government as 3^rd ^party funder' perspectives.

Data collection for the economic appraisal is integrated in the resource monitoring, process and outcome measures described above. On the cost side, pathway analysis and patient flowcharts will be used to fully specify all treatment activities (i.e. activity; probability of occurrence; no of times activity occurs; unit price). Resource costs to be included in the study (e.g. ICU bed occupation, general hospital bed occupation, complications & adverse events, tests & procedures, etc) will be fully specified in the economic evaluation protocol before the study commences. The appraisal will include sensitivity and uncertainty analysis (using the @Risk software) to explore the impact on cost-effectiveness results of uncertainty in cost and outcome data. Discounting will not be relevant to this study as the time horizon is less than one year.

### Adverse experiences

All adverse experiences either observed by the investigator or one of the clinical staff, or reported by the patient's parents/guardians spontaneously or in response to a direct question, that occur during the study period or a need for a change in therapy, will be evaluated by the investigator and noted in the adverse experience section of the patient's CRF. Events after the study period also thought to be due to a study intervention will be included.

A serious adverse event (SAE) is generally defined as any event that is fatal, life-threatening, permanently disabling, incapacitating or results in hospitalisation, prolongs a hospital stay or is associated with congenital abnormality, carcinoma or overdose.

Note: an event which is an expected and measured outcome of the trial can be treated as an adverse event rather than a serious adverse event (e.g. delayed discharge due to an infected IV site is an adverse event not an SAE).

#### Serious adverse events

For this trial, serious adverse events are the following:

• death during the study period

• aspiration pneumonitis associated with NG tube misplacement

• intrapulmonary infusion of fluid

• electrolyte disturbance causing seizure

• Any other event not mentioned above that is life threatening event or jeopardises the patient or requires medical or surgical intervention

### Reporting SAEs

SAEs need to be reported within 24 hrs by telephone to the local site investigator. The local investigator must report the SAE to their local ethics committee within 48 hours (or in accordance with local ethics committee regulations). The local investigator must also report SAEs to the principal investigators who will report the event to the Data Monitoring Committee chairman. The principal investigator will also report the SAE to all Ethics Committees and other regulatory bodies involved in the trial as per regional regulatory requirements.

SAEs will also be reported to the consultant of the child's treating team. The medical consultant will initiate appropriate management and inform the family if the family is not already aware of the event. This reporting is the responsibility of the site investigator.

### Limitations

This trial has a number of potential limitations. The interventions could not be blinded and clinician or researcher bias may influence clinical decision making. However, with the documented rates of use of each intervention approaching 50% - documented prior to the trial ^23 ^-this is unlikely to significantly impact on the outcome. Further, the trial is attempting to assess the use of the interventions in the real world clinical environment where these biases are in play, so the ability to translate the trial results to actual clinical situations may actually be improved. The environment where this study is being performed has low use of ancillary medications (e.g. bronchodilators and steroids) in bronchiolitis patients[23]. Although subgroup analysis would be desirable, the sample size to achieve this would not be possible within our study setting. The trial excludes children under 8 weeks of age and any results will not be generalisable to this population. No scoring system is used in this trial to determine severity, this remaining a clinical decision representing the real clinical environment, but may inhibit reproduction of the results.

## Discussion

The study commenced recruitment in mid 2009. Currently over 190 patients have been randomised. Patient recruitment is expected to be completed by December 2011.

## Abbreviations

IV: intravenous; NGT: nasogastric tube; IVR: intravenous rehydration; NGR: nasogastric rehydration; CPAP: continuous positive airway pressure; sd: standard deviation; SAE: serious adverse event; PREDICT: Paediatric Research in Emergency Departments International Collaborative

## Competing interests

The authors declare that they have no competing interests.

## Authors' contributions

EO and FEB were responsible for identifying the research question, and contributing to drafting of the study protocol. SD, MS, AD, JA, MB, DK, TT and JN have all contributed to the development of the protocol and study design, as members of the research team. EO was responsible for the drafting of this paper, although all authors provided comments on the drafts and have read and approved the final version. EO, for the PREDICT research network, takes responsibility for the manuscript as a whole.

## Pre-publication history

The pre-publication history for this paper can be accessed here:

http://www.biomedcentral.com/1471-2431/10/37/prepub

## Supplementary Material

Additional file 1 **Inclusion and exclusion criteria**. This file is a list of the inclusion and exclusion criteria used for the study Click here for file

Additional file 2 **Guidelines for changing interventions**. This file is the guidelines, supplied for the managing clinicians, on when patients should be changed from Nasogastric to Intravenous intervention, and from intravenous to Nasogastric intervention. Click here for file
