# Supplementary material for: A prospective randomised trial comparing nasogastric with intravenous hydration in children with bronchiolitis (protocol) The comparative rehydration in bronchiolitis study (CRIB)
Source: BMC Pediatr. 2010 Jun 1;10:37. doi: 10.1186/1471-2431-10-37 (PMC2903564; doi:10.1186/1471-2431-10-37)
Supplement: Additional file 1 — Inclusion and exclusion criteria. This file is a list of the inclusion and exclusion criteria used for the study [file 1471-2431-10-37-S1.DOC]

Inclusion criteria:

- Age
  - more than 8 weeks corrected for prematurity (as defined by the American Academy of Pediatrics) ^[1, 2]^
  - and less than 12 months,
- Symptoms of bronchiolitis
  - (as defined by the American Academy of Pediatrics)^[1, 2]^ - symptoms and signs of respiratory distress (tachypnoea, recessions, nasal flaring, and cyanosis) associated with symptoms of a viral respiratory tract infection (cough, runny nose, blocked nose).^[1, 2]^
- Requiring non oral fluid replacement
  - (defined as*:* history of reduced oral intake in at least the last 6 hours and reduced urine output - fewer or less full wet nappies - or signs of dehydration such as tachycardia (in the absence of fever), dry mucous membranes, decreased skin turgor, or sunken fontanelle).

Exclusion criteria:

- Chronic respiratory, neurologic or cardiac disease.
- Choanal atresia.
- Severe dehydration (needing IV resuscitation).
- Need for immediate ventilatory support.
- Patients with oxygen saturation below 90% despite 3L/min of oxygen via nasal cannula, or 50% oxygen via a head box or oxygen tent.
- Reasons for needing IV access other than bronchiolitis (investigations, medication).
