# Supplementary material for: A prospective randomised trial comparing nasogastric with intravenous hydration in children with bronchiolitis (protocol) The comparative rehydration in bronchiolitis study (CRIB)
Source: BMC Pediatr. 2010 Jun 1;10:37. doi: 10.1186/1471-2431-10-37 (PMC2903564; doi:10.1186/1471-2431-10-37)
Supplement: Additional file 2 — Guidelines for changing interventions. This file is the guidelines, supplied for the managing clinicians, on when patients should be changed from Nasogastric to Intravenous intervention, and from intravenous to Nasogastric intervention. [file 1471-2431-10-37-S2.DOC]

### Conditions for crossover from NGR to IVR:

- Infants who cannot maintain their SpO_2_ above 90% despite a maximum of 3L/min of oxygen via nasal cannula or 50% oxygen via head box or oxygen tent.
- Or need for ICU admission or ventilatory support (including CPAP) – defined as rising PaCO_2_ (at least >60 mm Hg), and increasing work of breathing, or apnoeas.
- Or vomiting repeatedly (>6 in any 6 hour period).
- Or deterioration in circulatory parameters requiring IV fluid bolus (severe tachycardia for age, reduced capillary refill (>3 seconds), with or without hypotension for age)
- Or parental insistence

**Conditions for crossover from IVR to NGR**

- Unable to place IV
- Or crying inconsolably and needing feeding
- Or parental insistence
